# Influence of Magnesium Ion Binding on the Adenosine Diphosphate Structure and Dynamics, Investigated by ^31^P NMR and Molecular Dynamics Simulations

**DOI:** 10.1021/acs.jpcb.4c02118

**Published:** 2024-09-10

**Authors:** Kelsey Anne Marr, David E. Korenchan, Alexej Jerschow

**Affiliations:** † Department of Chemistry, New York University, 100 Washington Square E, New York, New York 10003, United States; ‡ Martinos Center for Biomedical Imaging, Massachusetts General Hospital, 149 13th St, Charlestown, Massachusetts 02129, United States

## Abstract

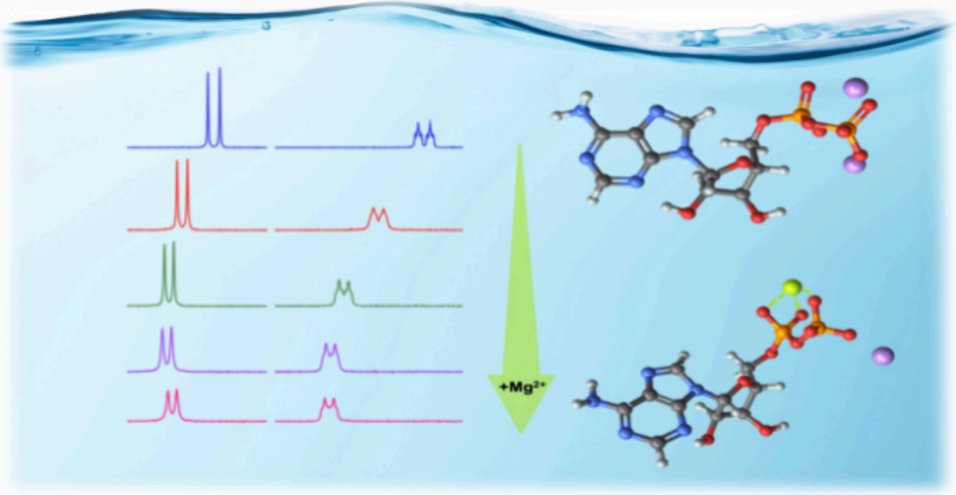

Magnesium (Mg^2+^) is the most abundant divalent cation in the cell and is essential to nearly every biochemical reaction involving adenosine triphosphate (ATP) and its lower energy counterpart, adenosine diphosphate (ADP). In this work, we examine the solution dynamics of ADP at different concentrations and record the changes thereof due to the presence of Mg^2+^ ions. Relaxation and diffusion experiments were performed on a range of ADP solutions with increasing magnesium concentration. The most significant changes of both relaxation and diffusion behaviors are observed when adding Mg^2+^ up to 0.5 ADP equivalent (eq), with most of the changes complete at 1 eq. Molecular dynamics simulations also show a significant structure introduced by Mg^2+^ with very stable pyramidal coordination with the phosphate oxygens. A more extended structure found in the presence of Mg^2+^ is consistent with the experimental slowing of diffusion and an increase in the spin–lattice relaxation rate. We do not observe direct evidence of aggregation in solution, although translational diffusion is slowed down significantly at higher concentrations (while solvent diffusion remains constant).

## Introduction

Magnesium is one of the most abundant cations in both eukaryotic and prokaryotic cells, playing a pivotal role in stabilizing negatively charged nucleotides during enzymatic reactions.^1^ The majority of these enzymatic reactions regulate metabolism with adenosine triphosphate (ATP) and its lower energy counterpart, adenosine diphosphate (ADP). Biological energy expenditure is paid when a high energy ATP molecule cleaves into a lower energy ADP and an inorganic phosphate molecule. These adenosine nucleotides are rarely seen without stabilizing magnesium cations in enzymatic environments, which is why the ATP/ADP-Mg^2+^ complex is considered the “biologically active” entity rather than the nucleotide alone.^2,3^ For these reasons, the interactions between magnesium ions and adenosine nucleotides are of great interest.

The structures of ATPases, the family of enzymes that catalyze the hydrolysis of ATP and ADP, are almost always crystallized with ADP-Mg^2+^ complexes in active or allosteric sites; however, there is no constant formation between the ADP and the Mg^2+^ as a singular unit with a uniform structure.^4^ For example, the F1-ATPase structure (PBD 1H8E) has three ADP-Mg^2+^ substrates within its subunits with each magnesium ion in a different orientation with respect to the phosphate groups on the corresponding ADP.^5^ In two of the subunits, the Mg^2+^ ion appears to associate with both phosphate groups equally, and the third subunit’s Mg^2+^ ion is associated only with the terminal β-phosphates. Regardless of the method by which these protein structures are studied, the same types of formations between ADP and Mg^2+^ are found.

The stability constants for ion binding to ADP, including Mg^2+^ binding, have been determined from pH titrations, potentiometric curves, and resin competition, all finding similar values.^6−8^ These results were also used to determine whether there was little interaction between Mg^2+^ and the adenosine ring of the nucleotide. ^1^H NMR further supported this finding and showed that there was little interaction between the cation and the adenosine base.^9^ Association constants of the adenosine base series (AMP, ADP, and ATP) have been determined by ^31^P NMR chemical shifts; these results led to the commonly seen magnesium chelate-style ring ADP-Mg^2+^ structure wherein one oxygen from each of the α- and β-phosphate groups complexes with the ion.^10,11^

The stability constants for ADP with and without Mg^2+^ cations present also pose another source of complexity, π-π interactions, leading to self-association. ADP, as a nucleotide, can self-associate due to its aromatic adenosine ring. Equilibrium constants for self-association have been determined by NMR, and ADP was reported to aggregate due to self-association.^3,12^ It was also reported that Mg^2+^ ions significantly influenced the self-stacking behavior by partially neutralizing the gathering phosphate groups.^13^ While the crystal structures of ADP in solution with K^+^ show some interaction of the cation with both the phosphate groups and water, there is no structural evidence of self-assembly by π-π interactions.^14,15^

The major aim of this project is to elucidate the nature of Mg^2+^-ADP binding and its role in modulating the structure, mobility, and potential aggregation. Here, we describe results from ^31^P NMR relaxation measurements along with diffusion experiments and molecular dynamics (MD) simulations, which together provide insights into the role of Mg^2+^ in changing the ADP solution structure.

## Materials and Methods

Adenosine-5′-diphosphate disodium salt, magnesium chloride salt, and Trizma base were bought from Sigma-Aldrich. Deuterium oxide (99.9 atom %) was purchased from Cambridge Isotope Laboratories. Three sets of ADP solutions were made, each with a standard amount of ADP of concentrations 10, 20, and 30 mM in D_2_O. Each of the solutions was given increasing half-equivalents of magnesium ions. For each ADP concentration, five samples were prepared to include magnesium equivalents of 0 (none added), 0.5, 1, 1.5, or 2; from here on, the abbreviation “eq Mg” is used to define the equivalents of Mg^2+^ to ADP concentration. The buffered pH of 8.5 was used as it is well above ADP’s p*K*_a_ values, therefore having predominantly the ionization state −3 as expected in biological systems.^9^ Then, 3 mM Trizma base was added as this buffer has little interaction with Mg^2+^ ions.^9,17^ Samples were buffered to pH 8.5 using HCl and NaOH D_2_O solutions. Chemical shifts were calibrated to the naturally occurring inorganic phosphate peak within each sample.

NMR experiments were performed using a Bruker 11.7T spectrometer equipped with a broadband direct-observe CryoProbe, tunable to ^1^H and ^31^P. Measurements were taken at 298 K. The 90° pulse durations for both nuclei were calibrated each day before any measurements were taken. All recycle delays were set to 8 s, which is over five times longer than any measured T_1_ values for ^31^P nuclei. Appropriate recycle delays for ^1^H were used (>35 s) when applicable. A standard 90° pulse-acquire sequence was used with the following optimized parameters for ^31^P nuclei: 16k data points, 48 ppm spectral width, and 16 scans. Processing parameters included line broadening at 1 Hz. Such parameters were maintained for T_1_ measurements by inversion recovery (T1IR standard Bruker sequence), T_2_ measurements, and self-diffusion experiments. Gradients for DOSY experiments were calibrated with the 1D STEGP1S1D pulse program and used in the STEGP1S diffusion pulse sequence. Traditional T_2_ relaxation measurements were made using a spin–echo sequence, often with the Carr–Purcell–Meiboom–Gill (CPMG) pulse train. When this sequence was used for ^31^P ADP, however, this exponentially decaying signal was alternating consistently between positive and negative phases over the course of the experiment. To circumvent this issue, the Periodic Refocusing of J-Evolution by Coherence Transfer (PROJECT) sequence was applied to the ADP samples.^18^ This pulse sequence uses a 90° pulse along the *y*-direction in a double spin–echo sequence, thus forming what is known as a “perfect echo.” The 90° pulse leads to refocusing of the J-modulation.

Self-diffusion coefficients from DOSY spectra were found by fitting to the Stejskal–Tanner equation:
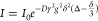
1where *I* is the observed intensity, *I*_0_ is the reference value (unattenuated intensity), *D* is the diffusion coefficient, γ is the gyromagnetic ratio of the observed nucleus, *g* is the applied gradient strength, δ is the duration of the applied gradient, and Δ is the diffusion time, which is the time between the starts of the defocusing and the refocusing gradients.^19^

MD simulations of ADP with both magnesium and sodium ions were performed to compare to experimental results using AMBER at 300 K using an NPT ensemble. MD simulations in Amber20 were performed similar to the approach in previous studies,^20−22^ which is restated here with minor modifications: ADP was parametrized using ESP charges obtained from Gaussian 16 with B3LYP/6-31G(d). The polyphosphate parameters of Homeyer et al.^23^ and Steinbrecher et al.^24^ and the monovalent ion parameters of Joung and Cheatham,^25^ and the Li/Merz multivalent 12-6 ion parameters were used.^26,27^ Any remaining parameters were obtained from the GAFF2 force field. A TIP4PEW water box with ∼35Å dimensions was used. Minimization was performed in 5000 steps, the timesteps were 1 fs throughout, and the final isothermal/isobaric ensemble (NPT, 300 K, 1 bar) 4 ns production run had a time step of 1 fs. Coordination distances between the ions and atoms on the phosphate were extracted along the trajectories using the Visual Molecular Dynamics (VMD) program. An *ab initio* calculation of chemical shift anisotropy tensors was performed by geometry optimization of the triply negatively charged ethyl pyrophosphate (as a proxy for ADP) with an implicit water solvent using B3LYP/6-31G(d) followed by chemical shift tensor calculations using B3LYP/aug-cc-pVTZ, also with an explicit water solvent.

## Results and Discussion

**Figure 1:**
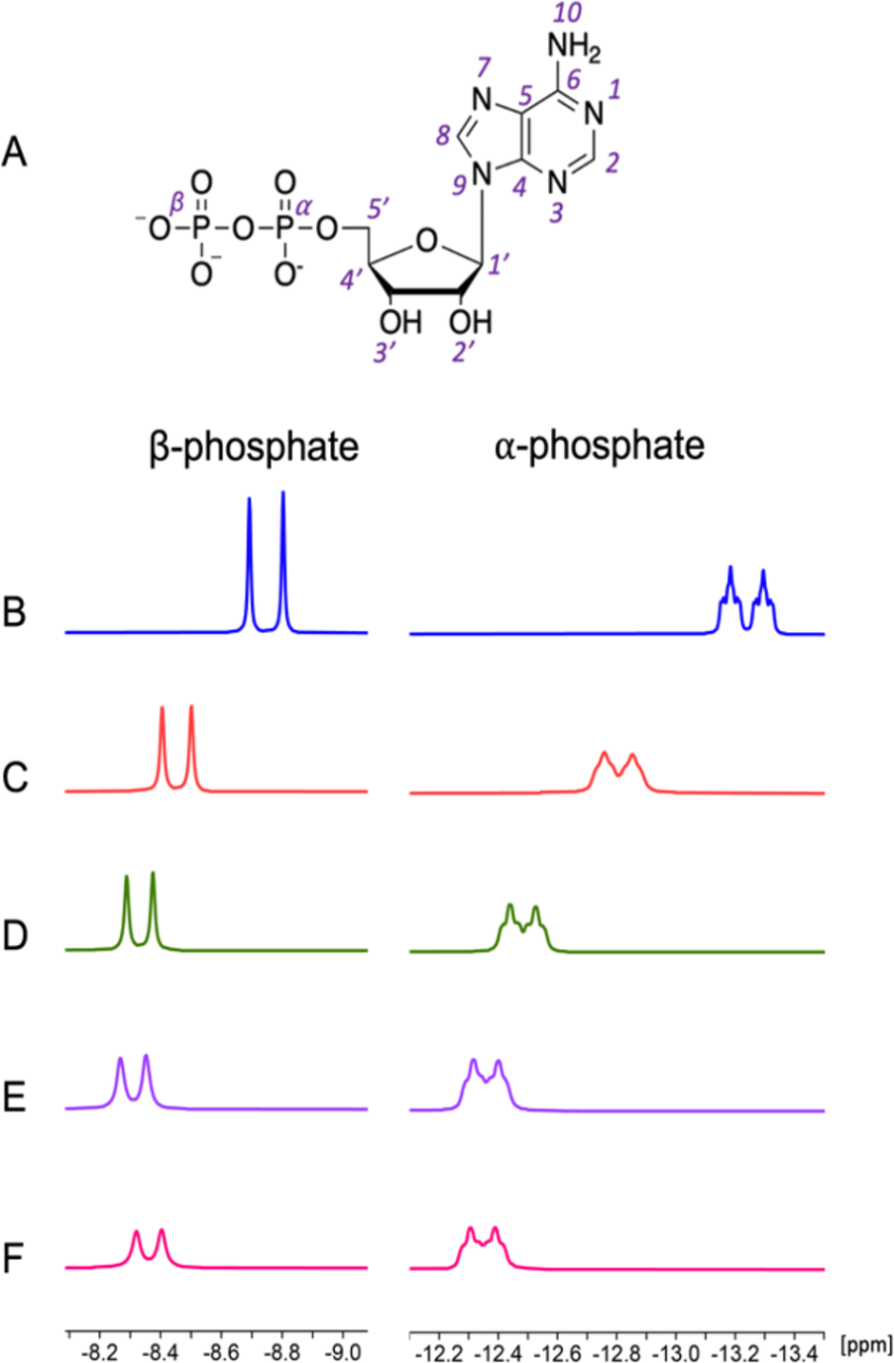
(A) Labeled ADP structure. ^31^P NMR spectra of ADP with (B) no Mg^2+^, (C) 0.5 equivalent (eq) Mg^2+^, (D) 1 eq Mg^2+^, (E) 1.5 eq Mg^2+^, and (F) 2.0 eq Mg^2+^.

The ^31^P spectra of samples without magnesium had narrower resonances, and the four-bond ^31^P-^1^H *J*-coupling of the α peak was clearly resolved (Figure 1). Peak broadening was also observed in the ^1^H spectra of the hydrogens at C-5′, the ribose carbon closest to the α-phosphate, as expected. Both the peak line width and the difference in chemical shifts between the α- and β-phosphates decreased with increasing cation concentration.

The ^31^P chemical shift difference between α- and β-phosphate peaks without any magnesium ions was 4.3 ppm, while at 1 equivalent (eq) Mg, the chemical shift difference dropped to 4 ppm and stayed within the 3.9–4 ppm range at any higher cation equivalence ratios (Table 1). The α- and β-phosphate multiplets in Figure 1 shifted downfield by maximum shifts of 0.89 and 0.44 ppm, respectively, upon addition of Mg^2+^. The larger magnitude chemical shift changes for the α-phosphate multiplet are in line with the binding models observed in MD calculations, which we discuss below. In addition, the ^31^P-^31^P *J*-couplings between the α- and β-phosphorus atoms decrease with increasing amounts of Mg^2+^ ions but change a little for different ADP concentrations (Table 2).

**Table 1:** ^31^P Chemical Shift Differences (Δδ) in ppm between α- and β-Phosphate Groups with Increased Equivalents (eq) of Mg^2+^

	**eq Mg**^**2+**^				
**[ADP] (mM)**	**0**	**0.5**	**1**	**1.5**	**2**
10	4.38	4.18	4.06	3.99	3.95
20	4.36	4.22	4.06	3.97	3.92
30	4.38	4.26	4.06	3.97	3.9

**Table 2:** ^31^P-^31^P *J*-Coupling in Hz between α- and β-Phosphate Groups with Increased Equivalents (eq) of Mg^2+^

	**eq Mg**^**2+**^				
**[ADP] (mM)**	**0**	**0.5**	**1**	**1.5**	**2**
10	22.6	19.5	17.7	17.3	17.2
20	22.5	19.4	17.6	17.1	16.9
30	22.5	19.4	17.5	17.0	16.7

The progressive downfield shift of the ^31^P α- and β-phosphate multiplets suggests that there exists a Mg^2+^-induced fast chemical exchange between ADP-Mg^2+^ binding states. By contrast, slow exchange would be indicated by the appearance of two distinct multiplets, one for each binding model following Δω ≫ *k*_ex_ with Δω being the chemical shift difference between α- and β-phosphate peaks (in radians per second) and *k*_ex_ as the exchange rate (in inverse seconds). When moving into the fast-exchange regime, these two distinct multiplets converge into a single peak, where Δω ≪ *k*_ex_. Since we only observe one peak per phosphate, it can be concluded that our ADP-Mg^2+^ system is in the fast-exchange regime. Consequently, the overall exchange rate can be estimated to be greater than at least five times the chemical shift dispersion, i.e., *k*_ex_ > 5500 s^–1^, based upon the chemical shift dispersion of the α- and β-phosphate multiplet.

To extract the proportions of the different exchange pools, we first modeled the exchange as a two-state system: a “free” state characterized by an absence of the ADP-Mg^2+^ interaction and a “bound” state in which Mg^2+^ interacted with the ADP molecule. Assuming that (1) the “free” state exhibits a ^31^P chemical shift in the absence of chemical exchange (δ_f_) equal to that observed with 0 eq Mg^2+^ added, for a given concentration, (2) the “bound” state exhibits a ^31^P chemical shift without exchange (δ_b_) equal to the largest downfield shift observed for the given concentration, and (3) the chemical system is in the fast-exchange regime (i.e., δ_b_ – δ_f_ ≪ *k*_1_ + *k*_–1_, where *k*_1_ and *k*_–1_ are the forward and reverse (pseudo)first-order exchange rates, respectively), we used the following equation to calculate the proportions of “free” and “bound” ADP (*p*_f_ and *p*_b_ = 1 – *p*_f_, respectively) using the observed chemical shift (δ_obs_):^28^
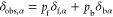
2

**Figure 2:**
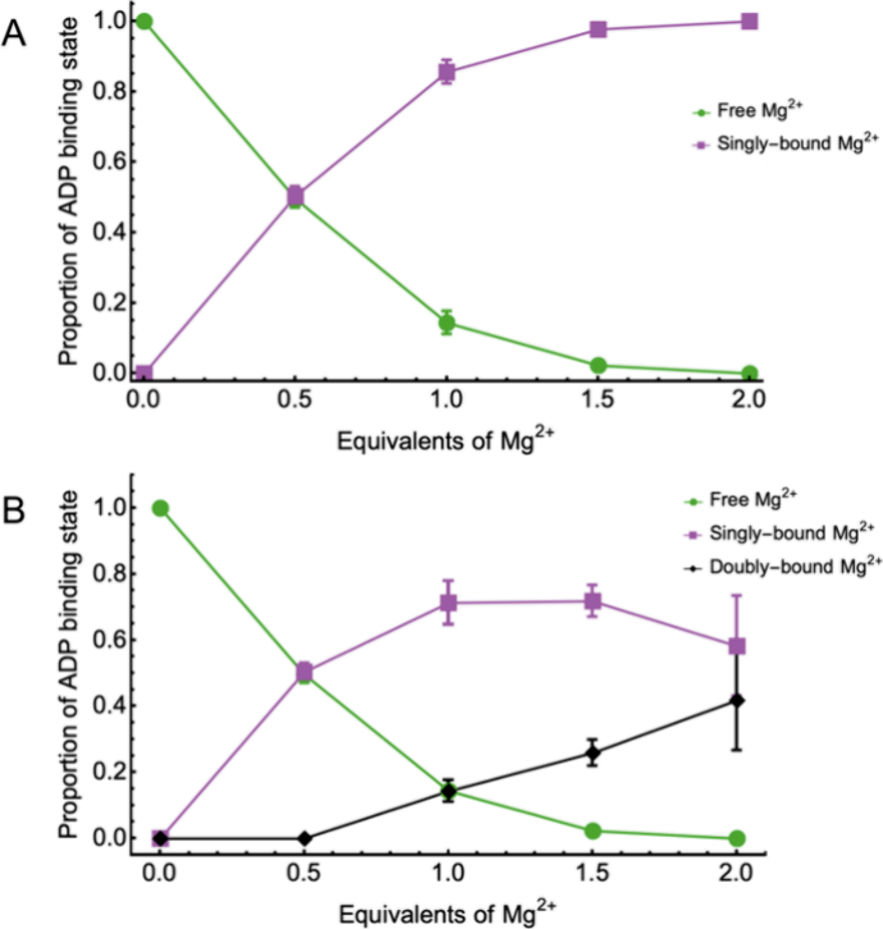
Modeled proportions of “free” ADP and “bound” ADP-Mg^2+^ complex using the ^31^P chemical shift as a function of eq Mg^2+^ added, using (A) the two-state system calculated by eq 2 or (B) the three-state system calculated by eqs 3, 4, and 5.

The results of the analysis are shown in Figure 2A. Note that the data represent values across three ADP concentrations (10, 20, and 30 mM); the δ_f_ and δ_b_ were determined at each individual concentration as the average shift of all the peaks in the multiplet and then used to calculate *p*_f_ and *p*_b_. The α-phosphate multiplet analysis matched well with our two-site exchange model: nearly 50% binding was observed at 0.5 eq Mg^2+^ regardless of the ADP concentration, and the proportions asymptotically approached their maximum/minimum values as the amount of Mg^2+^ increased.

Unlike the α-phosphate chemical shifts, the β-phosphate chemical shifts did not monotonically increase but rather increased below 1–1.5 eq Mg^2+^ and then decreased slightly at higher eq Mg^2+^ (see Figure 1). This observation suggests that a two-state model would not fit the β-phosphate shifts well. We therefore proceeded to model the β-phosphate chemical exchange as a process involving three distinct chemical states. In addition to a state where ADP binds a single Mg^2+^ cation, we added another state representing the sequential binding of a second Mg^2+^ cation:
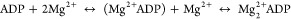
3

We assume that this second Mg^2+^ binding event affects only the β-phosphate shift, whereas the first involves both the α- and β-phosphate shifts. In the three-site model, the observed chemical shift of the β-phosphate is the population-weighted average of the chemical shifts of the three binding states: “free” ADP, “singly-bound” ADP, and “doubly-bound” ADP:
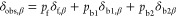
4with
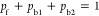
5where *p*_b1_ + *p*_b2_ = *p*_b_. Note that our experimental data suggest that δ_f,β_ < δ_b2,β_ < δ_b1,β_. This binding model requires solving for six unknown parameters, three of which are constant (δ_f,β_, δ_b1,β_, and δ_b2,β_) and three of which vary with the eq Mg^2+^ (*p*_f_, *p*_b1_, and *p*_b2_). The chemical shift of the free ADP (δ_f,β_) was easily determined to equal the chemical shift without Mg^2+^ added (0 eq). We also set the free ADP population at each ADP concentration (*p*_f_) to equal the value determined by fitting the α-phosphate NMR chemical shifts. This approach left us with four unknown parameters (two populations that vary with eq Mg^2+^ and two constant chemical shifts) and two equations. To fit the remaining variables, we made the following assumptions:

1. We considered the amount of doubly-bound ADP at 0.5 eq Mg^2+^ to be negligible (i.e., *p*_b2_(0.5eq) = 0). We justified this assumption based on the claim that 0.5 eq Mg^2+^ provides too little Mg^2+^ to promote a second Mg^2+^ to bind after the first cation. This approach allowed us to solve for the chemical shift of the singly-bound ADP (δ_b1,β_).
2. We constrained the proportions of the singly- and doubly-bound ADP so as not to exceed the number of equivalents of Mg^2+^ added (χ_Mg_ = [Mg^2+^]/[ADP]):
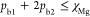
3. We then set the chemical shift of the doubly-bound ADP to have the minimum possible separation from the singly-bound ADP while fulfilling this constraint on the bound ADP proportions.

With these assumptions, one can solve for the bound-state proportions (*p*_b1_ and *p*_b2_) at each value of Mg^2+^ added, as reported in Figure 2B.

**Figure 3:**
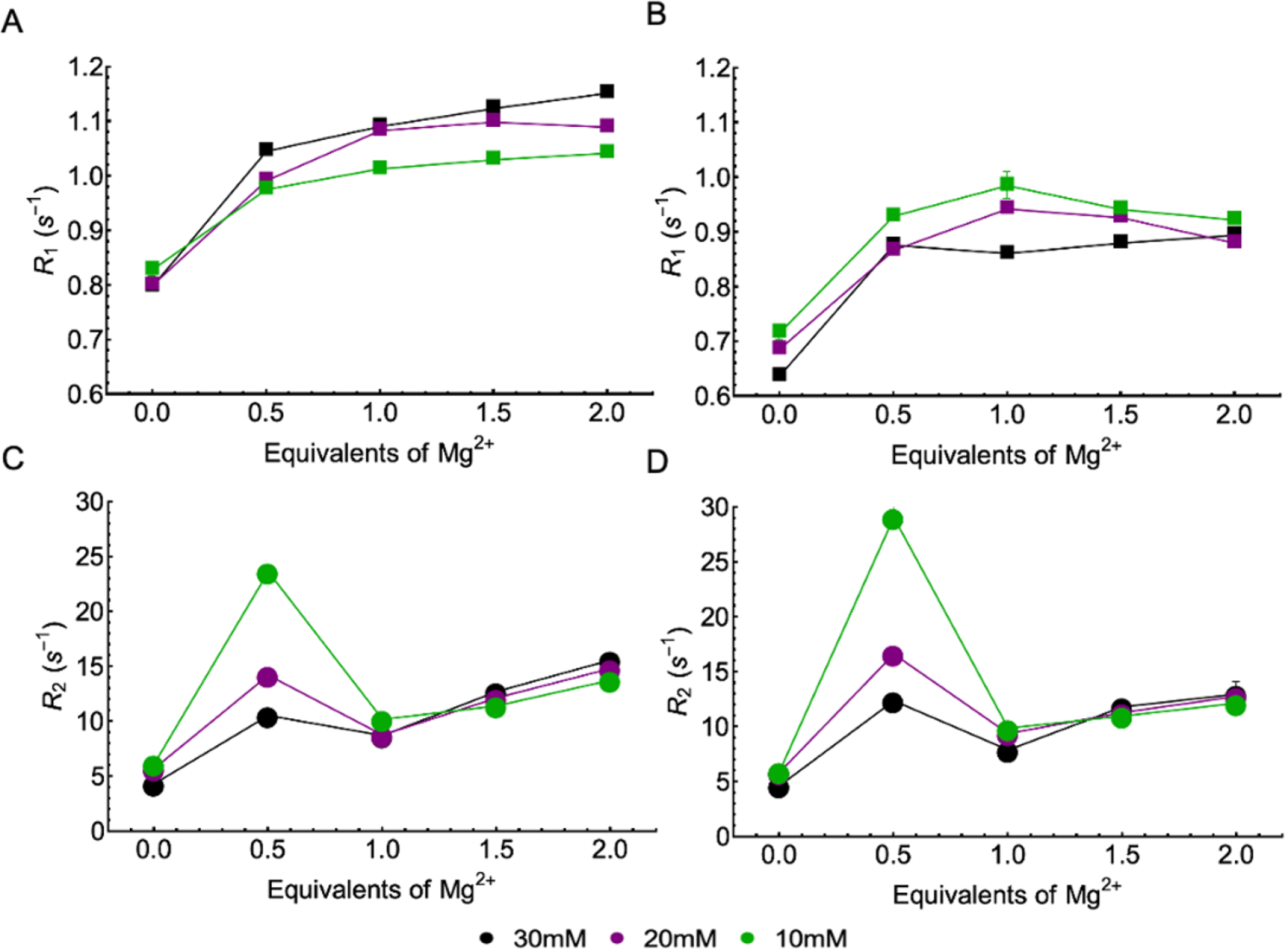
(A, B) ^31^P *R*_1_ relaxation rate constants of α- and β-phosphates, respectively, with increasing equivalents of magnesium ions. (C, D) ^31^P *R*_2_ relaxation rate constants of α- and β-phosphates, respectively, with increasing equivalents of magnesium ions.

Relaxation rates *R*_1_ =  and *R*_2_ =  were measured for both the α and β ^31^P peaks of all samples (Figure 3). In all curves, we observe the largest changes to occur when reaching 0.5 eq Mg^2+^ ions. Notably, after a large increase of *R*_1_ up to 0.5 eq, the following changes remain relatively small. Still, a further steady increase is observed for the α peaks with increasing Mg^2+^ content, while on average, a more plateau-like behavior is observed for the β peak. Also, the *R*_1_ values increased somewhat with increasing concentration above 0.5 eq for the α peaks. Interestingly, here again, the trend for the β peak is different as the values for 20 and 30 mM ADP begin to drop. These decreases of the β *R*_1_ values above 1 eq Mg^2+^ parallel the chemical shift movements already discussed above (seen in Figure 1D-F), which we see as a further indication that a two-site binding model is not sufficient to describe the β ^31^P data. Since the isotropic shift trend changes the direction at higher Mg^2+^ equivalents, it is reasonable to assume that additional Mg^2+^ binding or coordination to the available β oxygens could decrease the chemical shift anisotropy (CSA) tensor overall, which would explain the observed slight decrease in *R*_1_ rate constants.

The spin–spin relaxation rate constant (*R*_2_), by contrast, showed a steady increase with little influence, except at the 0.5 eq point. At 0.5 eq, we observe the *R*_2_ values to increase with ADP concentration. To ascertain that these results were not an incidental finding, each sample’s experiment was performed in triplicate for all data points. ANOVA analysis was used to determine that at each concentration, the *R*_2_ value at 0.5 eq Mg^2+^ was statistically different (*p* < 0.05) from the *R*_2_ values at all other equivalents. This 0.5 eq Mg^2+^ point represents a situation where the kinetic Mg^2+^ binding properties change rapidly. Consequently, we can also observe that whatever kinetic effects at play here are maximal at this point, and stable thermodynamic control is reestablished at 1 eq and beyond. This finding further supports the results from *R*_1_ measurements showing that 1 eq Mg^2+^ was sufficient to establish a significant and stable structural change of the ADP phosphates.

**Figure 4:**
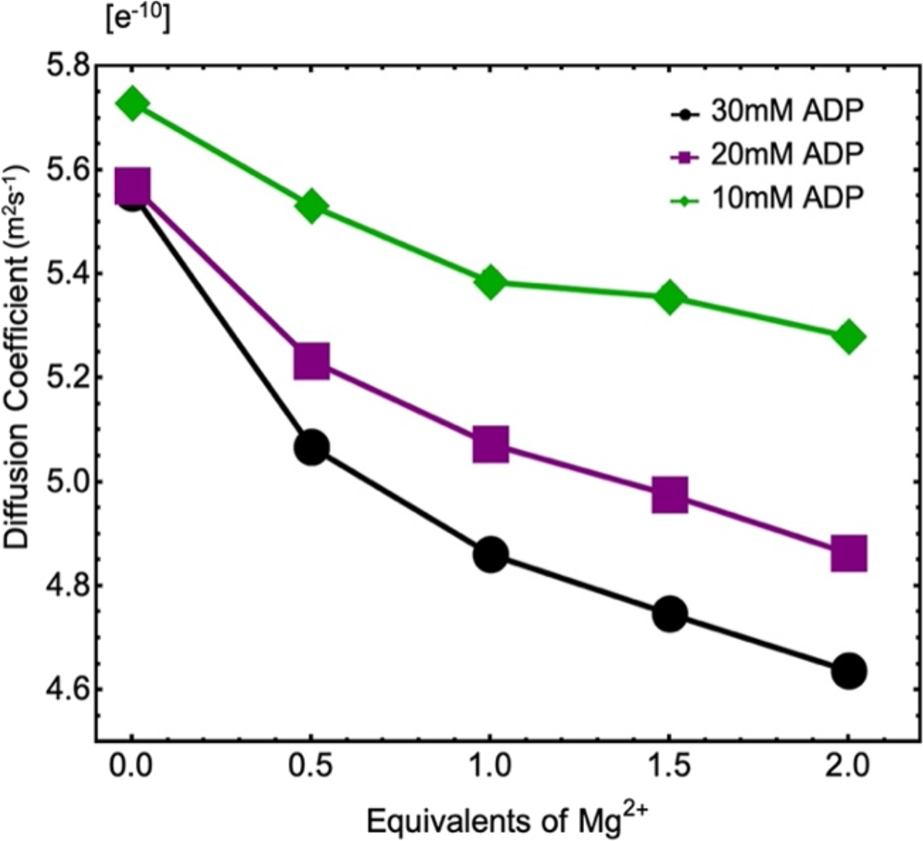
Diffusion constants of ADP:Mg^2+^ complexes from the DOSY experiments.

To identify potential aggregation, ^31^P DOSY experiments were performed to determine the translational diffusion coefficients of the various ADP solutions used in the relaxometry series. Figure 4 reports the average diffusion coefficients of the α and β ^31^P peaks. As could be expected, the diffusion coefficients decreased when the ADP concentration increased for any given Mg^2+^ concentration. Furthermore, we observed that the diffusion coefficient decreased significantly with an increasing Mg^2+^ concentration. The difference in ADP concentration seemed to hold less significance in both relaxation and diffusion measurements, in comparison to the change in Mg^2+^ equivalence. The ^1^H diffusion coefficient of the residual water peak in these samples remained nearly constant and matched the known self-diffusion coefficient of neat water.^29^

**Figure 5:**
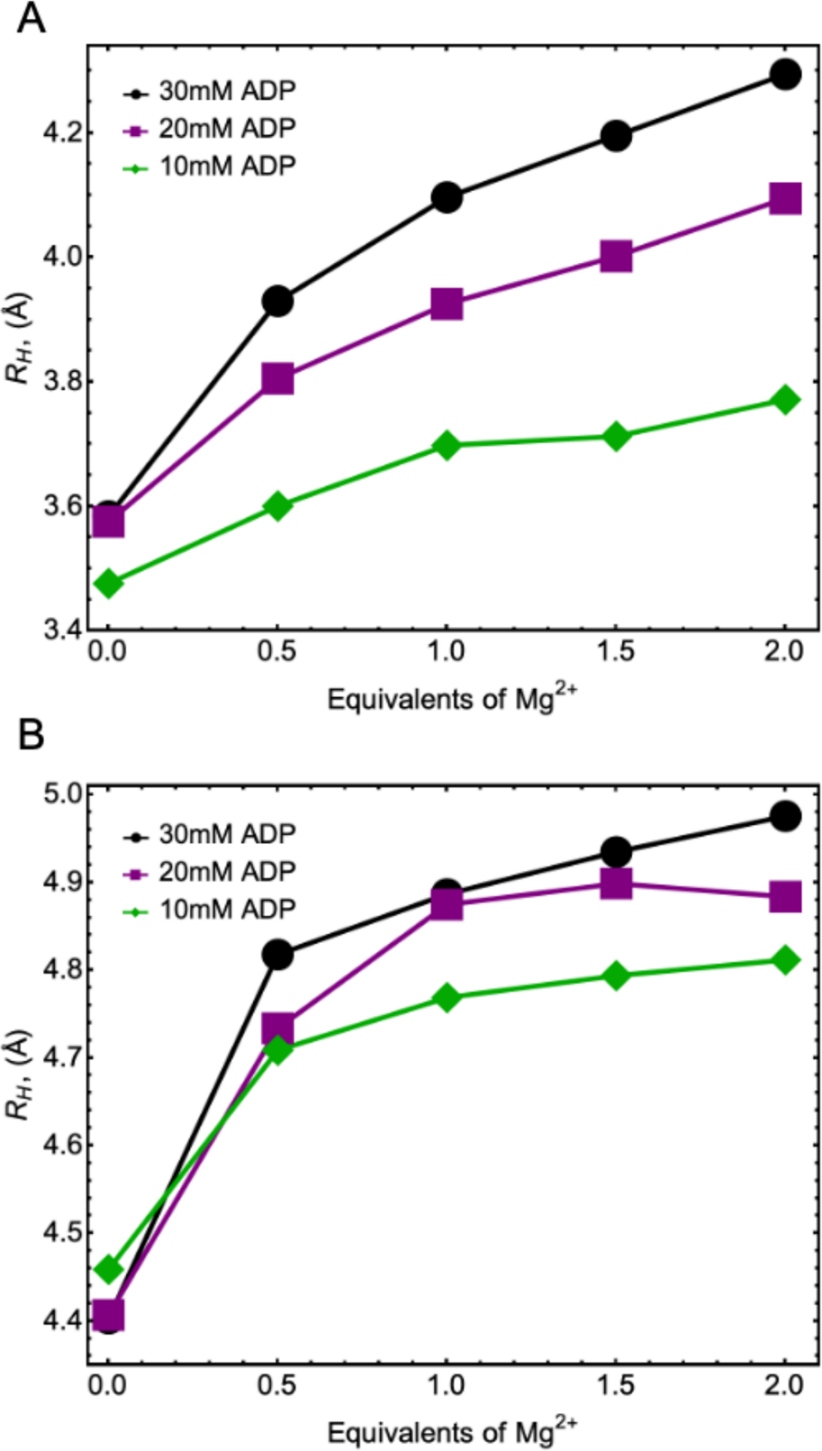
(A) Hydrodynamic radii of ADP:Mg^2+^ complexes determined from the diffusion coefficients via eq 6 and (B) hydrodynamic radii of ADP:Mg^2+^ complexes determined from ^31^P α *R*_1_ rates from eqs 7–9.

These diffusion coefficients were used to solve for the hydrodynamic radii, as shown in Figure 5. The hydrodynamic radii *R*_H_ shown here were determined via the Debye–Einstein equation:
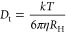
6where *k* is the Boltzmann constant, *T* the temperature, and η the viscosity of the solvent. It is important to note that the Debye–Einstein equation is strictly applicable only to spherical particles (of macroscopic dimensions) but is often found to provide good estimates for qualitative trends between different experimental parameters.

It is of further interest whether relaxation rates could also be used to estimate a hydrodynamic radius, but in this case, it is based on rotational diffusion.^16^ It is known that the major contribution to ^31^P *R*_1_ at high field for phosphates arises from chemical shift anisotropy (CSA) and it can be calculated by
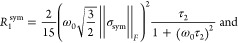
7
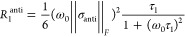
8for both the symmetric (sym) and antisymmetric (anti) portions of the CSA tensor. In this expression, ∥σ_sym_∥_*F*_ and ∥σ_anti_∥_*F*_ are the Frobenius norms of the respective CSA tensor components, ω_0_ is the Larmor frequency, and τ_2_ and τ_1_ are the second and first rank rotational tumbling correlation times, respectively.^21^ For this analysis, we chose the α ^31^P *R*_1_ data as they appeared to be better behaved and because this group is closely tethered to the bulky components of the molecule. From the *ab initio* calculation, we obtained ∥σ_sym_∥_*F*_ = 163 ppm for the α ^31^P. The antisymmetric component was very small by comparison and could be neglected. From the experimental values, it was hence possible to determine the correlation time τ_2_ with the help of eq 7. To obtain a representation comparable to the one obtained for *R*_H_ for the diffusion data, we used the expression^30^
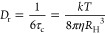
9

The results for *R*_H_ from both diffusion and relaxation are shown in Figure 5. We observe that the trends in *R*_H_ from both diffusion and relaxation are qualitatively the same, with the ones derived from translational motion showing a greater dispersion based on the ADP concentration and the ones derived from relaxation displaying larger radii. The radii extracted from relaxation data are close to what one could expect for these structures (especially when the molecule is not extended). The hydrodynamic radii further reflect contributions from local motion of the phosphate group. The large change in behavior up to 0.5 eq Mg^2+^ is more pronounced for the values derived from rotational motion, and the values do not increase much beyond that.

The relatively modest increases in hydrodynamic radii overall do not support the hypothesis of significant aggregation occurring as the concentration increases or even as Mg content increases. The insignificant changes of *R*_H_ derived from *R*_1_ further would indicate that the individual molecules rotate as their own units. On the other hand, it is reasonable to assume that ADP molecules may influence each other’s translational motion through long-range interactions as translational diffusion is a longer time-scale process. The control experiment of residual ^1^H diffusion showed that water diffusion remained relatively constant, which supports the notion that viscosity was not significantly affected by the presence of the ions and ADP in this concentration range (such changes are typically observed only at concentrations of about an order of magnitude higher).

**Figure 6:**
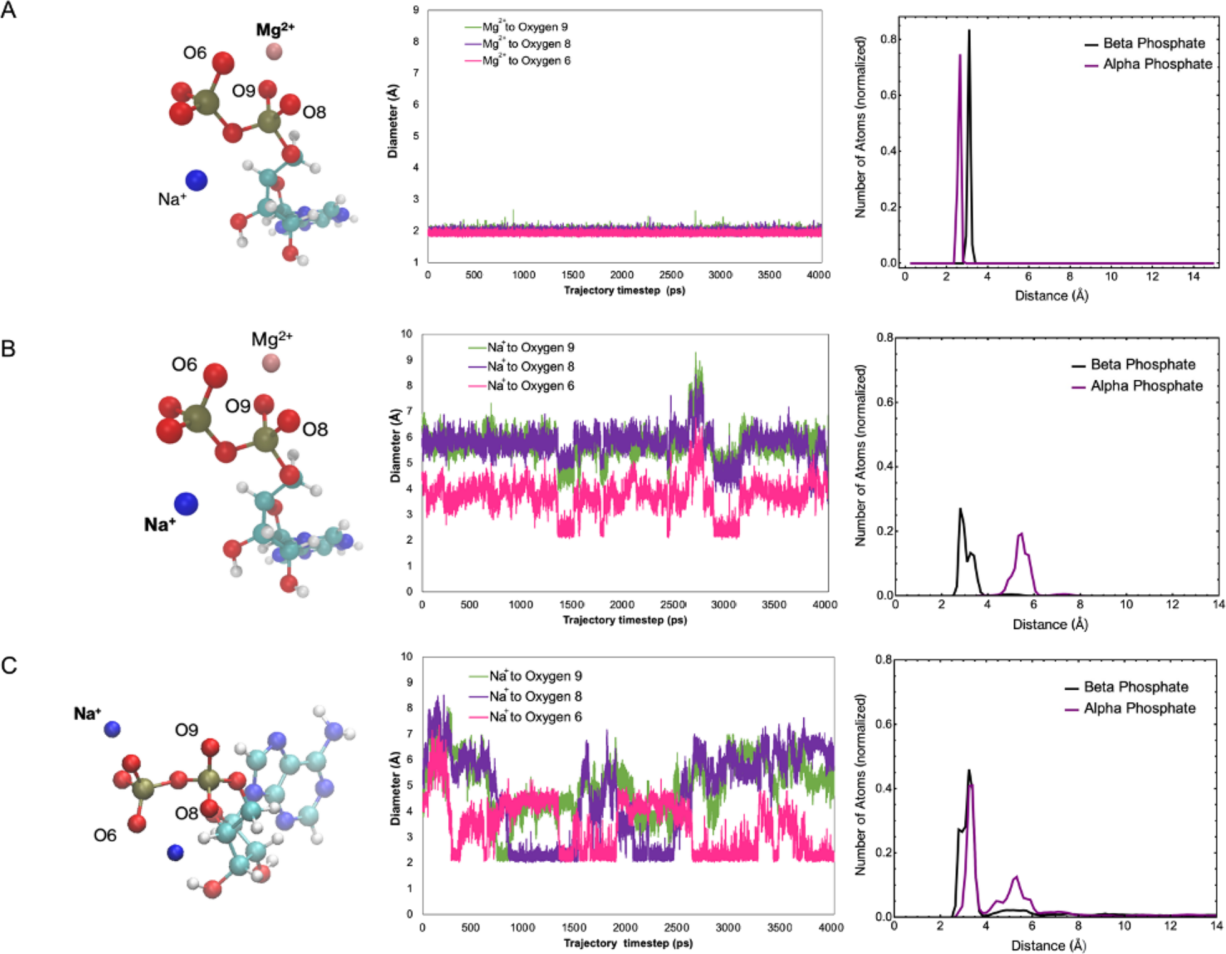
(A) Left to right: visual of MD simulation of one ADP, one Na^+^, and one Mg^2+^ in a solvated water box; distance between Mg^2+^and O6 located on ADP’s β-phosphate and O8 and O9 on the α-phosphate across the MD trajectory; radial distribution functions of Mg^2+^ w.r.t. the β- and α-phosphorus of the same MD simulation. (B) Left to right: same simulation as (A) with emphasis on the Na^+^ ion; distance between Na^+^ and O6 located on ADP’s β-phosphate and O8 and O9 on the α-phosphate across the MD trajectory; radial distribution functions of Na^+^ w.r.t. the β- and α-phosphorus. (C) Left to right: visual of MD simulation of one ADP and three Na^+^ in a solvated water box; distance between Na^+^ and O6 located on ADP’s β-phosphate and O8 and O9 on the α-phosphate across MD trajectory; radial distribution functions of Na^+^ to β- and α-phosphorus.

We now examine the potential structural changes induced by the presence of Mg^2+^ with the help of MD simulations. The simulations show that when Mg^2+^ is associated with ADP, it forms a triangular pyramidal structure with two oxygens from the α-phosphate and a single oxygen from the β-phosphate as its base. To evaluate the stability of the structure, we examine the distances between the magnesium ion and the three oxygens across the MD trajectory. The distances are all on average approximately constant at approximately 1.9 Å, as seen in Figure 6. By contrast, when examining the association of sodium ions with the phosphates, we find that the ions move around and are much less strongly coordinated with the oxygen atoms. It is of note that when a single Mg^2+^ ion is present, order is automatically increased, and the remaining sodium ion also keeps more constant distances to the oxygen atoms. The sodium–oxygen coordination, however, appears at larger length scales, as seen by comparing Figures 6B and 6C.

Here again in the radial distribution functions (RDFs), it is obvious that the Mg^2+^ coordination is the most stable one based upon the narrow distribution of coordination distances. Sodium is more loosely coordinated, with the order slightly increased in the presence of Mg^2+^. As expected, in the presence of one Mg^2+^, the Na^+^ ions are more closely coordinated with the β-phosphorus atom than with the α, whereas the opposite is the case for the Mg^2+^ ion. This difference in coordination to the α- and β-phosphorus atoms is also seen in ADP crystal structures with both sodium and potassium ions.^14,15^ The sharp peaks in the RDF curves for Mg^2+^ are clearly again an indication of the relative stability of the Mg^2+^ coordination.

Based on these results, we suggest that the slowdown of both rotational and translational motion with the addition of Mg^2+^ cations (and consequently also an increase in the hydrodynamic radii) is due to the more rigid and more laterally extended structure of ADP in such situations.

We note that although MD simulations report that the protons (H28 and H29) on the carbon (C-5′) closest to the α-phosphate should be within 2.8 and 3.5 Å, respectively, we were unable to detect a {^31^P}-^1^H heteronuclear Overhauser spectroscopy (HOESY) signal, which would be indicative of such proximity. This is likely due to the weak expected cross-relaxation rates at such distances for this nucleus pair. This absence of clear HOESY effects indicates that there does not appear to be an any more compact structure present experimentally than what is seen in the MD simulations.

## Conclusions

We present results of ^31^P and ^31^H NMR studies along with MD simulations, which provide clues for the Mg-ADP binding processes. In particular, we notice that Mg^2+^ ions, when bound to ADP, lead to a more extended structure than free ADP, which is evident in larger relaxation rates and slower diffusion. The hydrodynamic radii obtained from diffusion and relaxation show similar trends at different ADP concentrations, with diffusion showing a larger dispersion. We suggest that this finding is an indication of intermolecular interactions becoming stronger at higher concentrations. The relaxation-derived quantities, however, appear to indicate independent motion of the ADP units. MD studies show the very stable pyramidal binding of Mg^2+^ to phosphate oxygen atoms, while sodium is bound in a more diffuse way. The α-phosphate is coordinated with two oxygens and the β-phosphate with one oxygen to Mg^2+^, thus leaving two other oxygens for further ion coordination, for which chemical shift and relaxation changes seem to provide indications. In the presence of Mg^2+^, sodium binding also becomes more ordered.
